# Auditory Biofeedback Gait Training in Individuals With Chronic Ankle Instability: Protocol for a Randomized Controlled Trial

**DOI:** 10.2196/107775

**Published:** 2026-10-22

**Authors:** Danielle Marie Torp, Ji Yeon Choi, Alexandra Edison, Matthew Hoch, Kyle Kosik, Amelia Bruce Leicht, Ilana Patlan, Stacey Slone, Abbey Thomas, Jennifer Xu, Luke Donovan

**Affiliations:** 1 Department of Athletic Training and Clinical Nutrition, Sports Medicine Research Institute College of Health Sciences University of Kentucky Lexington, KY United States; 2 Dr Bing Zhang Department of Statistics University of Kentucky Lexington, KY United States; 3 School of Data Science Department of Applied Physiology, Health, and Clinical Sciences University of North Carolina at Charlotte Charlotte, NC United States; 4 Department of Applied Physiology, Health, and Clinical Sciences University of North Carolina at Charlotte Charlotte, NC United States

**Keywords:** external biofeedback, rehabilitation, motor learning, ankle sprain, biomechanics, joint health

## Abstract

**Background:**

Chronic ankle instability (CAI) develops in a large majority of patients with lateral ankle sprain (LAS), who often experience recurring injuries, residual feelings of instability, and abnormal neuromuscular patterns. Specifically, gait alterations are common in those with CAI and considered a contributing factor to poor long-term ankle joint health. Yet, there are no evidence-based gait-training protocols targeted to improve biomechanics, which may improve long-term outcomes related to ankle joint health and patient satisfaction.

**Objective:**

This study protocol introduces a progressive auditory biofeedback (ABF) gait-training program aimed to improve short- and long-term lower extremity biomechanics and talar cartilage outcomes and reduce the frequency of ankle-giving-way episodes and recurring injuries.

**Methods:**

This study is a multisite randomized controlled trial (RCT) with a target enrollment of 100 patients with CAI into a control group (n=50, 50%) or an ABF gait-training program (n=50, 50%). Both groups will complete a progressive 6-week (12-session) protocol comprising walking, ruck marching, and running on a treadmill. Lower extremity kinematics and kinetics, talar cartilage characteristics (via ultrasonography), self-perceived dysfunction and instability, and number of recurring sprains will be assessed prior to intervention, immediately after intervention, and 6 and 12 months postintervention. Finally, the frequency of ankle-giving-way episodes in the 6 months prior to intervention will be compared to the frequency of episodes at the 6- and 12-month time points to determine a longitudinal difference in ankle-giving-way episodes.

**Results:**

The study was funded in September 2024 and prospectively registered through ClinicalTrials.gov on March 10, 2025. The first participant was enrolled in May 2025. As of August 2026, 118 individuals had been recruited and 19 participants enrolled. Recruitment will remain open until September 2027, and study completion is expected in September 2028. Data processing is projected to continue through September 2028, with the final report published in June 2029.

**Conclusions:**

This study will be the first RCT of ABF gait training to assess the immediate and long-term effects on lower extremity biomechanics, ankle joint health, and patient-reported outcomes in patients with CAI. Results from this study will advance the knowledge of long-term joint health that may be associated with poor outcomes post-LAS.

**Trial Registration:**

ClinicalTrials.gov NCT06868316; https://clinicaltrials.gov/study/NCT06868316

**International Registered Report Identifier (IRRID):**

DERR1-10.2196/107775

## Introduction

Lateral ankle sprain (LAS) is among the most common musculoskeletal injuries across the general population, athletes, and active-duty service members, placing significant strain on both civilian and military health care systems [1-3]. Although often considered self-limiting, a significant proportion of individuals experience persistent symptoms post-LAS, including recurrent sprains, feelings of instability, and ankle-giving-way episodes [4]. The compilation of these symptoms characterizes chronic ankle instability (CAI), which can develop within 6 months after the initial LAS [5,6]. Beyond sustaining recurring injuries, patients with CAI report reduced levels of physical activity, a constant level of diminished joint health, compromised physical and mental health, and heightened fear avoidance beliefs [7-10].

In addition to persistent functional limitations, CAI is strongly associated with early onset ankle posttraumatic osteoarthritis (PTOA), with nearly 78% of patients demonstrating degenerative changes [11-14]. The onset of ankle PTOA occurs nearly 10 years earlier than the onset of idiopathic ankle OA, thus directing clinicians and researchers to develop early interventions aimed at preserving joint health [15]. The development of ankle PTOA is theorized to be accelerated by an altered biomechanical profile that developed post-LAS, which produces abnormal joint contact stresses [16,17]. Specifically, patients with CAI demonstrate greater inversion angles and a more laterally deviated center of pressure (COP) trajectory compared to healthy controls [18]. Evidence suggests that this lateralized gait pattern is associated with worse talar cartilage health outcomes in patients with CAI [19]. Furthermore, patients with CAI have more cartilage deformation after loading compared to healthy adults [20,21]. Collectively, the evidence suggests that the altered biomechanical profile may contribute to the early cartilage degeneration onset of ankle PTOA and worse long-term health-related outcomes in patients with CAI. Thus, there is a need for interventions to target the aberrant biomechanical pattern portrayed by patients with CAI in order to improve ankle joint health and lifelong impacts post-LAS.

Current rehabilitation for patients with CAI primarily follows an impairment-based approach, with robust evidence supporting intervention strategies to target deficits in postural control, range of motion, and strength [9,22-24]. This evidence suggests that interventions tied to these impairments can improve their associated deficit and patients’ self-reported function. Unfortunately, studies also show that improvements in the aforementioned impairments do not alter gait patterns; thus, the long-term impact on joint preservation remains in question [25,26]. Targeting gait biomechanics through external biofeedback represents a promising strategy to address these impairments by facilitating motor learning during dynamic locomotor tasks [27-29]. Among the available modalities, auditory biofeedback (ABF) allows real-time, task-specific feedback during locomotor activities without reliance on visual input [30,31]. In addition, ABF may be more advantageous in repetitive sagittal plane activities, such as walking [32]. Despite encouraging preliminary findings, previous external biofeedback gait-training studies in CAI have been limited by small sample sizes and minimal long-term follow up, restricting the confidence [27]. Accordingly, there is a need for a progressive, adequately powered gait-training intervention that directly targets gait impairments and evaluates both biomechanical and joint health outcomes to determine long-term improvements in overall quality of life and ankle-related joint health.

Therefore, the purpose of this multisite randomized controlled trial (RCT) is to assess the effectiveness of a 6-week gait-training program with ABF, compared with a control program, to improve lower extremity biomechanics, ankle joint health, and patient-reported outcomes (PROs). Our central hypothesis is that implementing ABF gait training will improve lower extremity biomechanical function and ankle joint health and reduce the occurrence of ankle-giving-way episodes and future ankle sprains. The study will be guided by the following specific aims:

- Aim 1: Determine whether a 6-week gait-training program with ABF improves lower extremity biomechanics compared to a control condition in participants with CAI.
- Aim 2: Determine whether a 6-week gait-training program with ABF reduces talar cartilage deformation compared to a control condition in participants with CAI.
- Aim 3: Determine whether a 6-week gait-training program with ABF reduces ankle-giving-way episodes and self-perceived severity of symptoms compared to a control condition in participants with CAI.

## Methods

### Research Design

This study will use a single-blind, multisite, parallel RCT design conducted at the University of Kentucky (UK) and the University of North Carolina at Charlotte (Charlotte). Participants will report to the UK Sports Medicine Research Institute or the Charlotte Biodynamics Research Laboratory to complete outcomes preintervention (baseline) and at three time points (immediately, 6 months, and 12 months) postintervention. Each data collection session will comprise (1) lower extremity biomechanics during walking, ruck marching, and running; (2) talar articular cartilage resting thickness and deformation via ultrasonography; and (3) ankle-giving-way episodes and PROs of CAI symptom severity.

The information reported in this protocol for methodology followed SPIRIT (Standard Protocol Items: Recommendations for Interventional Trials) 2025 recommendations (Multimedia Appendix 1) [33], and descriptions of the intervention procedures followed the TIDieR (Template for Intervention Description and Replication) guide (Multimedia Appendix 2) [34]. This clinical trial was registered and first posted in the United States National Library of Medicine through ClinicalTrials.gov (NCT06868316) on March 10, 2025. Table 1 outlines key details of the registered trial. Final results will be updated on the registry as well as disseminated through conference abstracts, manuscripts, and final reporting to the funding sponsor.

**Table 1:** Key details of the registered trial.

Data category	Information
Primary registry; trial ID	ClinicalTrials.gov; NCT06868316
Date of registration in primary registry	March 10, 2025
Secondary IDs	98018, CDMRP-OR230135 (other grant/funding number: Department of Defense)
Source(s) of monetary or material support	Congressionally Directed Medical Research Programs
Primary sponsor	Congressionally Directed Medical Research Programs
Secondary sponsor(s)	N/A^a^
Contact for public queries	Author DT
Contact for scientific queries	DT
Public title	Auditory Biofeedback Gait Training in Individuals With Chronic Ankle Instability: Protocol for a Randomized Controlled Trial
Scientific title	Optimizing Retention on Duty in Patients with Chronic Ankle Instability Using Auditory Biofeedback Gait Training: Multisite Randomized Controlled Trial
Country of recruitment	United States
Health condition(s) or problem(s) studies	Ankle injuries, ankle sprains
Intervention(s)	Active comparator: ABF^b^ and control gait training. Six weeks of ABF gait training will be delivered. Participants are expected to complete two supervised sessions each week.
Date of first enrollment	May 2025
Target sample size	100
Recruitment status	Recruiting ongoing
Primary outcome(s)	Gait biomechanics (time frame: 12 months) Ultrasound for ankle joint health Severity of CAI^c^-related symptoms (time frame: 12 months) Frequency of ankle-giving-way episodes (time frame: 12 months)

^a^N/A: not applicable.

^b^ABF: auditory biofeedback.

^c^CAI: chronic ankle instability.

### Ethical Considerations

Ethical approval was obtained from UK’s Institutional Review Board (#98018), with reliance agreements and ethical approvals provided by Charlotte. This protocol was reviewed and approved by the Office of Human Research Oversight of the US Army Medical Research and Development Command Office of Human and Animal Research Oversight. This study was supported by the Congressionally Directed Medical Research Programs, Peer-Reviewed Orthopedic Research Program (grant HT9425-24-1-0882). Aside from human subjects’ research protection review, the funding sponsor did not play any direct role in the study design; data collection, management, analysis, or interpretation; manuscript writing; report drafting; or decision-making to submit for publication. Trial monitoring will be conducted quarterly by the principal investigators and study coordinators, with oversight by the Office of Human Research Oversight. Written informed consent will be obtained from all participants prior to enrollment by an authorized member of the research team. All participants will demonstrate understanding of the study prior to enrollment After baseline testing, intervention specialists will open a sealed opaque envelope that contains the group assignment for the corresponding subject ID. Separate investigators will be used for intervention delivery and outcomes assessment. Investigators collecting data at baseline and immediately postintervention will remain blinded to group assignments until the end of the study period. Similarly, licensed rehabilitation specialists administering the interventions will be blinded to performance on outcome measures during testing sessions. All data will be entered and stored in a custom-made Research Electronic Data Capture (REDCap) database to facilitate restricting data access and preserve blinding.

### Participants

In total, 100 men and women with CAI will be recruited from the surrounding area of UK (n=50, 50%) and Charlotte (n=50, 50%) through printed flyers, social media posts, and word of mouth. Enrolled participants will be randomized in blocks of 10 (1:1 allocation ratio) into a control group (n=50, 50%) or an ABF group (n=50, 50%); the sequences at each site will be separate and include randomizations for up to 60 participants each.

The inclusion criteria mostly align with the International Ankle Consortium’s (IAC) position statement on criteria for including participants with CAI in studies [35]. To be included, participants must be physically active male or female adults aged 18-45 years, with a history of at least one ankle sprain at least 4 weeks prior to enrollment and at least two ankle-giving-way episodes in the past 3 months. An ankle sprain is defined as an incident in which the rearfoot is inverted or supinated, resulting in swelling, pain, and time loss or modification of normal function for at least 1 day. An ankle-giving-way episode is described as an incident in which the rearfoot suddenly rolls, feels weak, or loses stability; however, the individual does not sustain an ankle sprain and can continue with their normal function. In addition, participants must answer yes to ≥5 questions on the Ankle Instability Instrument (AII) and ≥11 questions on the Identification of Functional Ankle Instability (IdFAI) instrument. The AII and IdFAI are reliable and valid instruments for assessing perceived ankle instability in patients with CAI [36,37]. In cases of bilateral CAI, the limb with the highest IdFAI score will be selected as the involved limb. Moderate aerobic physical activity in the past month will be determined by participants reporting a score of ≥4 on the National Aeronautics and Space Administration (NASA) Activity Instrument and indicating they can run for at least 20 consecutive minutes.

Exclusion criteria include (1) an ankle sprain sustained in the prior 4 weeks or a lower extremity neuromusculoskeletal injury or fracture, other than an ankle sprain, in the past 12 months; (2) a history of surgery in the lower extremities; (3) a history of neurological disease, vestibular/visual disturbance, or any other pathology that would impair sensorimotor performance; (4) current participation in a formal ankle joint rehabilitation program; (5) concussion in the past 12 months; (6) cardiovascular, metabolic, or renal disease or signs and symptoms of such conditions; and (7) instructions by a medical provider to refrain from vigorous activity. Healthy volunteers were not accepted.

These criteria deviate from the IAC’s recommendations in two instances: (1) The first LAS is not required to be >12 months prior to the study, and (2) the most recent sprain is not required to be >3 months prior to the study. These adaptations will enhance the clinical applicability of the intervention, as individuals who present with feelings of instability and perceived dysfunction would still be seeking treatment.

### Testing Sessions

Assessments of ankle-giving-way episodes, PROs, ultrasonography, and biomechanics will be completed in identical sessions at baseline, immediately postintervention, and after 6 and 12 months (Table 2).

**Table 2:** Summary of outcome measures and when they will be collected.

Outcome measures	Baseline	Immediately postintervention	At 6 months	At 12 months
Lower extremity kinematics	Yes	Yes	Yes	Yes
Plantar pressure profiles	Yes	Yes	Yes	Yes
Talar cartilage characteristics	Yes	Yes	Yes	Yes
Ankle-giving-way episodes	Yes	No	Yes	Yes
Self-perceived symptom severity	Yes	Yes	Yes	Yes

#### Ankle-Giving-Way Episodes

The ability of ABF gait training to reduce the frequency of ankle-giving-way episodes, compared to a control group, will be determined through self-reported occurrences in the past 6 months. Participants will report the number of ankle-giving-way episodes over the past 6 months at baseline and at 6 and 12 months postintervention through an electronic survey. At the 6- and 12-month time points, participants will also be asked to report any new LASs through this survey. During each survey, the same definitions used at the time of screening and enrollment for ankle giving way and LAS will be provided to maintain consistency with participant recall.

#### Patient-Reported Outcomes

The ability of ABF gait training to diminish the severity of related symptoms, compared to a control group, will be determined through the Cumberland Ankle Instability Tool (CAIT) and the Foot and Ankle Disability Index (FADI). Higher scores on these tools are indicative of better function and less severity of symptoms. CAIT will be completed at baseline, immediately postintervention, and at 6 and 12 months postintervention to assess perceived ankle instability. CAIT includes nine items that capture the severity of perceived ankle instability by capturing the degree of difficulty individuals have due to their ankle while performing various physical activities [38]. FADI will be used to assess function limitations during activities of daily living and sports-related activities [39] and will be assessed at baseline, immediately postintervention, and at 6 and 12 months postintervention. The inclusion of FADI was selected over the common Foot and Ankle Ability Measure to include pain-related outcomes.

#### Ultrasonography

The ability of ABF gait training to alter talar cartilage parameters, compared to a control group, will be determined. B-mode ultrasonography will capture the talar articular cartilage in a resting state and after a loading protocol. Changes in thickness and cross-sectional area between resting and loaded will be assessed. Immediately after they provide consent, participants will sit for 30 minutes on a standard treatment table with their legs fully extended to limit the effect of previous weight-bearing activity on the talar cartilage resting thickness volume [40]. After the offloading period, the participants will sit with their back flat against the wall, the knee of the involved limb flexed at 90°, and the foot flat on the treatment table. A 12 MHz linear ultrasound probe (ACUSON Redwood) will be positioned transversely between the medial and lateral malleoli and adjusted until the talar cartilage appears maximally reflected [41]. Three images of the talar cartilage will be obtained and averaged to assess the talar cartilage at rest (unloaded). Next, participants will complete the biomechanical gait analysis described later and then will be immediately repositioned on the treatment table in accordance with their unloaded arrangement. Subsequently, three more images of the talar cartilage will be obtained and averaged for assessment (loaded).

#### Biomechanical Assessment

Participants will be fitted with 1 mm thick in-shoe pressure insoles (Pedar with Plidar; Novel Electronics, Inc) placed into neutral laboratory shoes (Gel-Excite 9, Asics America Corporation). The pressure insoles connect to a transmitter fastened to the dorsum of the shoes, which communicates with a computer database via Bluetooth. Each participant will be fitted with seven inertial measurement units (IMUs with accelerometer ±160 m/s^2^, gyroscope 34.91 rad/s, and magnetometer ±1.9×10^–4^ Tesla of full-range scale; MTw Awinda, Xsens Technologies BV). Sensors will be secured via straps to the lumbar spine at the L5 level, bilaterally on the lateral thighs, anteromedial shanks, and dorsum of the feet. The insoles and IMUs will be calibrated according to the manufacturer’s instructions. The Awinda and Pedar systems will be synchronized using a Pedar Sync device, which will trigger a transistor-transistor logic pulse in Awinda software, and the pulse will be saved alongside the Pedar pressure data.

Lower extremity gait biomechanics will be assessed on a treadmill (Woodway USA Inc) under three conditions in the following order: walking, ruck marching, and running. At baseline, participants will self-select their pace for each condition, which will be kept consistent across testing and rehabilitation sessions, and each participant will get 2 minutes to adjust their speed under each condition to find their target speed. For walking, participants will determine the speed by a pace that will enable them to *walk comfortably*. For running, participants will find a pace they perceive they can *comfortably sustain for at least 20 minutes*. For ruck marching, participants will the speed by a pace that will enable them to *walk comfortably with this load*. The rucksack weight will be standardized to 20% of each participant’s body weight based on the guidelines for optimal military performance thresholds for combat operations [42]. At the end of each 15-minute condition, participants will be asked to rate their perceived exertion using the Borg 6-20 scale [43].

Participants will walk, run, and ruck-march on the treadmill for 15 minutes under each condition. A 30-second trial of biomechanical data will be recorded at 3, 6, 9, 12, and 14.5 minutes to obtain a comprehensive gait analysis. For each 30-second trial, the middle 10 steps will be extracted, processed in the native software for each system, and averaged across trials.

### Gait-Training Intervention

Beginning within 7 days of baseline testing, both control and ABF groups will complete 12 gait-training intervention sessions over a 6-week period supervised by a licensed health care provider (e.g., certified athletic trainer or physical therapist). All participants will wear standard neutral athletic shoes (Gel-Excite 9) and wireless in-shoe force insoles (MLP Loadsol Pros) during every intervention session for standardization of testing procedures. The in-shoe force sensors will be equipped with three force sensors located over the heel, the medial foot, and the lateral foot and will connect to an Apple iOS app (Loadsol-S; Novel Inc) that enables real-time measurement of regional and total forces throughout the intervention. Each intervention session will last ~30-45 minutes. First, participants will walk for 5 minutes wearing neutral shoes fitted with the in-shoe force sensors to allow the sensors to stabilize and improve calibration accuracy [44]. After stabilization, the sensors will be calibrated according to the manufacturer’s guidelines [44]. Next, participants will complete a standardized 5-minute warm-up; the ABF group will receive ABF during the warm-up (see the *Auditory Biofeedback Intervention* section). After warm-up, participants will complete the designated 15-minute intervention condition for that session (walking, ruck marching, or running). Both groups will follow the same intervention condition progression: walking in sessions 1-4, ruck marching in sessions 5-8, and running in sessions 9-12. The starting pace for each condition will be the self-selected pace determined at baseline testing. Environmental progressions will be introduced to both control and ABF groups. However, the ABF group will only progress through the levels if the 90% adherence threshold is met (see the *Auditory Biofeedback Intervention* section). Each condition (walking, ruck marching, and running) will comprise four sessions corresponding to four levels (Table S1 in Multimedia Appendix 3):

- Level 1 will consist of the 15-minute designated condition at the baseline speed, with the treadmill incline set to 0%.
- At level 2, the treadmill speed will increase by 10% and the treadmill incline kept at 0%.
- At level 3, the treadmill incline will increase by 3%, whereas the treadmill speed will return to the baseline speed.
- At level 4, the treadmill incline will remain at 3%, and the treadmill speed will increase by 10%.

Participants will be encouraged to complete the intervention without disruption; however, requests for a break (rest, water, bathroom, etc) will be granted by pausing the intervention and resuming when participants are ready.

#### Control Intervention

The control group will be instructed to “walk, ruck-march, and run comfortably” for 15 minutes of each intervention session but will not be provided with any form of biofeedback. The group will progress through levels 1-4, as described before, for each condition regardless of performance (Table S2 in Multimedia Appendix 3). The parameters for the control group have been selected to account for any benefits that may be attributed to the addition of physical activity.

#### ABF Intervention

ABF will be provided during warm-up to allow familiarization before the 15-minute intervention begins. The biofeedback range will be established at session 1 of each condition after sensor stabilization and calibration. Before warm-up, participants will “walk, ruck-march, and run” naturally at their baseline speed for ~1 minute while a 30-second trial of force data is recorded through the force insoles. The middle 10 steps will be chosen to compute the average peak force generated by the lateral sensor. The biofeedback threshold will be set by calculating a 20% reduction in the average lateral peak force. A 30% reduction (floor) will also be calculated and used as the minimum threshold limit allowed during the respective conditions; this 30% threshold has been established to prevent participants from attempting to overcorrect their gait pattern and minimize potential discomfort. At the beginning of each subsequent session, a similar 1-minute walking trial with a 30-second trial of force data will be conducted. The interventionist will again calculate the average peak force produced by the lateral sensor during the middle 10 steps. A new 20% threshold will be calculated from the new average and used as the biofeedback threshold for that session; however, if the new 20% threshold exceeds the original 30% threshold, the original floor threshold will be used for every subsequent session remaining in that condition.

Once the biofeedback thresholds are established, the values will be set into the Loadsol-S app (Figure S1 in Multimedia Appendix 3). The interventionist will begin collecting a live measurement of the insoles and selecting the lateral sensor of the involved limb to deliver ABF. Participants will be provided with the following instructions: “As you move around, you’ll notice a noise is being generated from the insoles. Walk around, shift your weight around, and try to figure out how the noise is being generated.” Participants will then be guided to shift their weight from side to side, front to back, and walking around. All instructions will be repeated to maintain an external focus of attention and refrain from providing any internally focused prompting. An internal cue will direct attention to each participant’s foot, particularly the lateral foot. The interventionist will only provide instructions that direct the participants’ attention to the noise being generated from the insoles [27,28]. Once participants acknowledge they understand how the noise is being generated, they will get on the treadmill and begin the stabilization and warm-up sessions. The ABF will remain “on” during each warm-up session. After participants walk for ~1 minute while the ABF is sounding, the interventionist will cue them to “walk in a manner that you do not hear the beep, but that is still comfortable” [30]. If a participant successfully follows the instructions, they should not hear the ABF. If they revert their gait to excessively load their lateral foot, the participant will again hear the ABF. The interventionist will not provide any further instructions to the participants on how to achieve the cueing but rather will only repeat the original cue to “walk in a manner that you do not hear the beep, but that is still comfortable.” At the end of each session, clinicians will instruct participants to continue walking in a manner that would not elicit the beep when they leave the laboratory and continue their normal activities. At the conclusion of the study, participants will be reminded again to continue walking in a way they would not elicit a beep in their normal activity.

Progressions through each level will be individually determined to ensure that each participant is continually challenged throughout the rehabilitation program. To progress to the next level, participants will be required to adhere to the biofeedback for a minimum of 90% of the steps taken during the 15-minute condition portion of each intervention session. After each intervention session, the interventionist will export data from the Loadsol-S app and pass it through a custom MATLAB (MathWorks) code that will determine the level of adherence. The custom code will calculate the number of peaks generated by the lateral sensor and the percentage of those peaks that are above and below the designated biofeedback threshold. If ≥ 90% of the peaks are below the threshold limit, the participants will progress to the next level at the subsequent session. If the adherence level is <90%, the participants will remain at the same level for the next session.

#### Data Reduction and Processing

Ultrasonography images will be exported and processed via ImageJ (National Institutes of Health). A region of interest will be selected by tracing the border of the talar cartilage using the “Polygon” tool in ImageJ. Cartilage thickness in the medial and lateral regions will be manually measured at the center of the medial and lateral regions (mm). The normalized talar cartilage cross-sectional area will be calculated by manually outlining the cartilage and dividing the length of that region to obtain a total, medial, and lateral normalized cross-sectional area (mm^2^). Each pixel within the region of interest will be assigned a grayscale value ranging from 0 (black) to 255 (white) arbitrary units, and echo intensity will be calculated as the mean grayscale value across the region of interest for the overall, medial, and lateral regions. Talar cartilage deformation will be calculated using Equation 1 [30].

% Deformation = [(μ_loaded_) – (μ_unloaded_) / (μ_unloaded_)] × 100

Lower extremity joint kinematics will be processed using MVN Analyze Pro (XSens by Movella). Joint angles and segment kinematics will be calculated with a sampling rate of 100 Hz, exported, and smoothed with a fourth-order Butterworth filter using a custom MATLAB script. Biomechanical outcomes will include frontal and sagittal plane peak joint angles at the ankle, knee, and hip; tibial angle velocities; and tibial accelerations. Primary outcomes of interest will include the ankle joint inversion angle and tibial acceleration.

The ability of ABF gait training to improve common aberrant lower extremity biomechanics, compared to a control group, will be determined. Joint angles and plantar pressure profiles will be assessed during walking, ruck marching, and running with IMUs and in-shoe pressure insoles. Gait kinetics will be processed using the Pedar system to create a plantar pressure profile. Kinetic outcomes will be collected at 100 Hz and will include peak pressure (maximal load in a specific area under the foot in one step, kilopascals [kPA]), maximum force (greatest total force the foot applied to the insole in one step, Newtons [N]), pressure-time integral (cumulative load of pressure over time in an area under the foot, kPA·s), and force-time integral (cumulative load of force over time, N·s) for nine regions of the foot: medial and lateral heels; medial and lateral midfeet; medial, central, and lateral forefeet; great toe; and lesser toes). The COP gait line will also be calculated via a custom MATLAB script, where the distance (mm) of the COP location from the medial border of the foot during stance is extracted and averaged in 10% increments to create a trajectory from initial contact to toe-off [45].

The study team will compare clinical and PROs collected at four time points (baseline, immediately postintervention, 6 months postintervention, and 12 months postintervention) between patients who undergo the ABF gait-training intervention and controls to determine whether ABF can further improve gait biomechanical and ultrasonographic measures of ankle joint health and reduce the rate of ankle-giving-way episodes during the 12 months postintervention.

#### Power Analysis

Using the effect size of 0.87 from preliminary data for aim 1 [30], an a priori power analysis assuming α=0.05 determined that 22 participants are needed per group to provide at least 80% power to detect a similar difference in biomechanical outcomes between groups at the 6-month time point. Using an effect size of 0.63 from preliminary data for aim 2 [30] and assuming α=0.05, 40 patients/group will provide 80% power to detect a difference in talar cartilage outcomes. Wright et al [46] reported that the percentage of patients with ankle-giving-way episodes decreased from 100% to 57% at 6 months postintervention. Assuming the same rate for the ABF group, 40 patients/group will allow us to see a 29.6% decrease compared to the control group with 80% power. For all three aims, all available data from each of the four time points will be used in the analysis to increase the power to see a significant difference. To allow for 20% attrition over the 12-month course of the trial, 50 patients will be enrolled in each group.

### Statistical and Data Analysis Plan

Continuous variables will be summarized with descriptive statistics using n (%), mean (SD), median (first and third quartiles), and range (minimum and maximum). Categorical variables will be described with counts and percentages. Change scores and percentage change scores will be summarized from baseline for follow-ups immediately postintervention and at 6 and 12 months postintervention; the primary outcome for all three aims is at 6 months. Numerical and graphical summaries will be provided overall and by randomized condition. Simple comparisons between groups will be performed using two-sample *t* tests for continuous variables and chi-square tests of independence for categorical variables. Comparison of means will be coupled with effect sizes with 95% CIs. A two-sided significance level of .05 will be used for all statistical tests. All analyses will be completed using SAS v9.4.

To achieve all three aims, each outcome will be analyzed using a repeated-measure generalized linear mixed model using all four time points, and appropriate correlation structures will be considered. Normality of the continuous outcomes will be assessed, and nonparametric statistical approaches will be used, if needed. For the binary aim 3, the logit link function will be used. The demographic characteristics of the ABF and control groups will be tested for imbalances, and any imbalance will be adjusted for in the final models. Potential confounders include baseline outcome values, demographic variables (eg, sex, age, height, and weight), prognostic indicators (eg, number of previous ankle sprains, number of ankle-giving-way episodes, and IdFAI score), and intervention compliance (% completed) and progression level.

The primary analysis will follow an intent-to-treat (ITT) approach and will be performed using all randomized participants regardless of follow-up data, where data for participants removed or lost to follow-up will be imputed using multiple imputations. Single imputation, such as last observation carried forward (LOCF), will be used to assess the sensitivity of results based on multiple imputations; additional analysis will also be conducted to compare groups as they are randomized with data as observed. LOCF and as-observed analyses should provide a conservative estimate of the effect, since the immediate impact of the intervention is expected to be more acute (LOCF). Participants who remain in the study may observe the most significant effects. An attrition analysis will compare demographics and outcomes measured at baseline between participants who complete follow-up and those who do not. Mechanisms for missing data will be investigated by comparing important covariates between participants with and without missing data at each time point. We will characterize these mechanisms as (1) missing completely at random (MCAR), (2) missing at random (MAR), or (3) missing not at random (MNAR). If the nature of our missing data is ignorable (either MCAR or MAR), we will use imputation methods, such as multiple imputation via chained equations, to handle dropouts. Ultimately, sensitivity analyses will be conducted to compare the results of our imputation methods with complete-case and available-data analyses. Furthermore, data on compliance measures will be captured in weekly intervention logs. A modified ITT analysis may also be conducted using an ITT randomization principle but limited to participants who achieve at least 75% of the intervention protocol across all 6 weeks.

## Results

The study was funded in September 2024 by the Congressionally Directed Medical Research Programs, Peer-Reviewed Orthopedic Research Program (grant HT9425-24-1-0882) and prospectively registered through ClinicalTrials.gov (NCT06868316) on March 10, 2025. The first participant was enrolled in May 2025, and as of August 2026, 118 individuals had been recruited and 19 participants enrolled. Participant recruitment is predicted to end in September 2027, with a final project date of September 2028. Data processing is projected to continue through September 2028, with the final report published in June 2029.

## Discussion

### Summary of Findings

This protocol of a multisite RCT describes a progressive ABF gait-training intervention to address gait impairments, ankle joint health, and PROs in patients with CAI. The study will evaluate the immediate and long-term effects of a 6-week progressive gait-training program that incorporates walking, ruck marching, and running, using comprehensive biomechanical outcomes, ultrasonographic outcomes, and PROs through 12 months postintervention. We hypothesize that ABF gait training will improve lower extremity biomechanics, show signs of improved talar cartilage outcomes, and reduce the occurrence of ankle-giving-way episodes compared to a control program.

The gait-training intervention described in this protocol expands the existing CAI literature, which has predominately focused on walking [27]. The progressive protocol will expose participants to multiple walking contexts (eg, faster speeds, uphill walking, and loaded walking), requiring them to adapt their gait in various situations. These task-oriented progressions are intended to promote adaptability and generalize motor learning beyond the clinic to navigate ecologically valid ambulation settings [47]. Although ruck marching is primarily a military task, loaded ambulation is common in daily life (eg, wearing a backpack, carrying groceries, or carrying children) and is known to alter kinematics and impact forces [48]. Accordingly, including loaded gait training (ruck marching) may improve real-world relevance of the intervention. The delivery of ABF in this protocol will use a commercially available tool that has also been a limitation of prior gait-training interventions using prototype devices [30,49] or expensive laboratory equipment [50]. The implementation of a widely available device allows clinicians to overcome practical barriers to adoption into clinical practice.

This protocol expands prior research by using a comprehensive biomechanical approach to quantify global gait adaptations by pairing kinematics (IMU system) and kinetics (Plidar system) for both limbs. In addition, our protocol will be the first ABF gait-training trial on CAI to determine whether learned gait changes persist beyond the initial acquisition period and can be maintained for up to 12 months postintervention. By pairing longitudinal biomechanical assessments with ankle joint health outcomes, this protocol provides a framework to test the biomechanical theory of PTOA development. Understanding the short- and long-term effects of gait training in patients with CAI will provide a vital context on rehabilitation success and the ability of clinicians to make an impact in long-term ankle joint health.

Rehabilitation research on ankle sprain is extensive, yet a substantial gap remains in interventions for the impairment domain of functional movement patterns associated with CAI [22,50]. We selected a randomized, parallel-group design with a dose-comparison control rather than a placebo or alternative biofeedback comparator because no standard-of-care gait-training intervention exists for this population [25,26]. Accordingly, our objective is to evaluate a progressive gait-training program that includes higher-demand locomotor activities beyond walking (ie, ruck marching and running) [27]. Including these higher-demand tasks is intended to improve ecological validity and test whether gait changes generalize to real-world conditions where speed, load, and fatigue may exacerbate CAI-related impairments.

### Conclusion

Collectively, this protocol aims to advance the current rehabilitation paradigm for gait training to identify the short- and long-term efficacy of a progressive program with multiple ambulatory modalities on a multimodal approach to patient-centered care.

## Data Availability

Data sharing is not applicable as no datasets were generated or analyzed during this study as of August 2026.

## Appendix

Multimedia Appendix 1 SPIRIT 2025 checklist of items to address in a randomized trial protocol. SPIRIT: Standard Protocol Items: Recommendations for Interventional Trials.

Multimedia Appendix 2 The TIDieR checklist. TIDieR: Template for Intervention Description and Replication.

Multimedia Appendix 3 Intervention progression for the ABF and control groups and sample depiction of ABF using the Loadsol-S iOS app. ABF: auditory biofeedback.

## Abbreviations

ABF: auditory biofeedback
AII: Ankle Instability Instrument
CAI: chronic ankle instability
CAIT: Cumberland Ankle Instability Tool
Charlotte: University of North Carolina at Charlotte
COP: center of pressure
FADI: Foot and Ankle Disability Index
IAC: International Ankle Consortium
IdFAI: Identification of Functional Ankle Instability
IMU: inertial measurement unit
ITT: intent to treat
LAS: lateral ankle sprain
LOCF: last observation carried forward
MAR: missing at random
MCAR: missing completely at random
MNAR: missing not at random
NASA: National Aeronautics and Space Administration
PRO: patient-reported outcome
PTOA: posttraumatic osteoarthritis
RCT: randomized controlled trial
SPIRIT: Standard Protocol Items: Recommendations for Interventional Trials
TIDieR: Template for Intervention Description and Replication
UK: University of Kentucky
