# Supplementary material for: Auditory Biofeedback Gait Training in Individuals With Chronic Ankle Instability: Protocol for a Randomized Controlled Trial
Source: JMIR Res Protoc. 2026 Oct 22;15:e107775. doi: 10.2196/107775 (PMC13740819; doi:10.2196/107775)
Supplement: Supplementary file 3 [file resprot_v15i1e107775_app3.docx]

**Multimedia Appendix 3**

**Auditory Biofeedback Group Intervention Progression Requirements**

Progressions will be individually determined to ensure each participant is being challenged while still benefiting during the entire rehabilitation program. The outline of the intervention sessions and progressions in Table S1. For a participant to progress in each task, they will have to adhere to the biofeedback during 90% of the 15-minute condition portion of each intervention session (Table S1). After each intervention session, the interventionist will export data from the Loadsol Pro iOS app and passed through a custom MATLAB code to determine adherence. The code will calculate the total steps taken and the number of steps within the biofeedback range (Figure S1, turquoise bar). For example, if a participant takes 2,000 steps during Session 2 and 1,900 of those steps fall within the biofeedback range (95%) then the participant will advance to the next progression during Session 3. However, if a participant only adheres to the biofeedback for 1,500 of the 2,000 total steps (75%) in Session 2, they will repeat the progression level during Session 3.

| **Table S1. Intervention Outline and Progressions for the Auditory Biofeedback Group** | | | | | |
| --- | --- | --- | --- | --- | --- |
|  | **Session** | **Condition and Time** | **Criteria for Progression** | **Progression** | |
| **Weeks 1 and 2** | 1 | Walk 25' | - | - | |
|  | 2 | Walk 10' | - | - | |
|  |  | Walk 15' | 90% Adherence to Biofeedback in Session 1 | Speed: +10% | Incline: 0% |
|  | 3 | Walk 10' | - | - | |
|  |  | Walk 15' | 90% Adherence to Biofeedback in Session 2 | Speed: Baseline | Incline: 3% |
|  | 4 | Walk 10' | - | - | |
|  |  | Walk 15' | 90% Adherence to Biofeedback in Session 3 | Speed: +10% | Incline: 3% |
| **Weeks 3 and 4** | 5 | Walk 10'  Ruck 15' | - | - | |
|  | 6 | Walk 10' | - | - | |
|  |  | Ruck 15' | 90% Adherence to Biofeedback in Session 5 | Speed: +10% | Incline: 0% |
|  | 7 | Walk 10' | - | - | |
|  |  | Ruck 15' | 90% Adherence to Biofeedback in Session 6 | Speed: Baseline | Incline: 3% |
|  | 8 | Walk 10' | - | - | |
|  |  | Ruck 15' | 90% Adherence to Biofeedback in Session 7 | Speed: +10% | Incline: 3% |
| **Weeks 5 and 6** | 9 | Walk 10'  Run 15' | - | - | |
|  | 10 | Walk 10' | - | - | |
|  |  | Run 15' | 90% Adherence to Biofeedback in Session 9 | Speed: +10% | Incline: 0% |
|  | 11 | Walk 10' | - | - | |
|  |  | Run 15' | 90% Adherence to Biofeedback in Session 10 | Speed: Baseline | Incline: 3% |
|  | 12 | Walk 10' | - | - | |
|  |  | Run 15' | 90% Adherence to Biofeedback in Session 11 | Speed: +10% | Incline: 3% |

**Control Group Intervention Progression**

The control group will progress in gait speed and treadmill incline on a standard timeline that will be consistent for every participant (Table S2)

| **Table S2. Intervention Outline and Progressions for the Control Group** | | | | |
| --- | --- | --- | --- | --- |
|  | **Session** | **Condition and Time** | **Environmental Progression** | |
| **Weeks 1 and 2** | 1 | Walk 25' | - | |
|  | 2 | Walk 10' | - | |
|  |  | Walk 15' | Speed: +10% | Incline: 0% |
|  | 3 | Walk 10' | - | |
|  |  | Walk 15' | Speed: Baseline | Incline: 3% |
|  | 4 | Walk 10' | - | |
|  |  | Walk 15' | Speed: +10% | Incline: 3% |
| **Weeks 3 and 4** | 5 | Walk 10'  Ruck 15' | - | |
|  | 6 | Walk 10' | - | |
|  |  | Ruck 15' | Speed: +10% | Incline: 0% |
|  | 7 | Walk 10' | - | |
|  |  | Ruck 15' | Speed: Baseline | Incline: 3% |
|  | 8 | Walk 10' | - | |
|  |  | Ruck 15' | Speed: +10% | Incline: 3% |
| **Weeks 5 and 6** | 9 | Walk 10'  Run 15' | - | |
|  | 10 | Walk 10' | - | |
|  |  | Run 15' | Speed: +10% | Incline: 0% |
|  | 11 | Walk 10' | - | |
|  |  | Run 15' | Speed: Baseline | Incline: 3% |
|  | 12 | Walk 10' | - | |
|  |  | Run 15' | Speed: +10% | Incline: 3% |

**Figure S1.** Sample depiction of the auditory biofeedback using the Loadsol-S iOS application. The biofeedback threshold (turquoise bar) is set accordingly, and if the force peaks of the lateral column (purple lines) exceed the threshold (indicated by the red circles) an audible “beep” would be generated. For force curves that do not exceed the threshold, there will be no sound made, indicating the participants are adhering to the biofeedback gait training.
